# Protocol for a randomized controlled multicenter trial assessing the efficacy of leuprorelin for severe polycystic liver disease: the AGAINST-PLD study

**DOI:** 10.1186/s12876-022-02142-y

**Published:** 2022-02-25

**Authors:** S. E. Aapkes, L. H. P. Bernts, A. P. van den Berg, M. van den Berg, H. Blokzijl, A. E. P. Cantineau, M. D. A. van Gastel, R. J. de Haas, P. Kappert, R. U. Müller, F. Nevens, R. Torra, A. Visser, J. P. H. Drenth, R. T. Gansevoort

**Affiliations:** 1 Department of Nephrology, University Medical Center Groningen, University of Groningen, Groningen, The Netherlands; 2 Department of Gastroenterology and Hepatology, Radboud University Medical Center, Nijmegen, The Netherlands; 3 Department of Gastroenterology and Hepatology, University Medical Center Groningen, University of Groningen, Groningen, The Netherlands; 4 Department of Obstetrics and Gynecology, University Medical Center Groningen, University of Groningen, Groningen, The Netherlands; 5 Department of Radiology, University Medical Center Groningen, University of Groningen, Groningen, The Netherlands; 6 Department II of Internal Medicine and Center for Molecular Medicine Cologne, Faculty of Medicine, University Hospital Cologne, University of Cologne, Kerpener Straße 62, 50937 Cologne, Germany; 7 Department of Gastroenterology and Hepatology, Universiteitsziekenhuis Leuven, Leuven, Belgium; 8 Department of Nephrology, Fundacio Puigvert, Barcelona, Spain; 9 Department of Applied Health Sciences, University Medical Center Groningen, University of Groningen, Groningen, The Netherlands

**Keywords:** Polycystic liver disease, GnRHa, Estrogen

## Abstract

**Background:**

In patients with severe polycystic liver disease (PLD), there is a need for new treatments. Estrogens and possibly other female sex hormones stimulate growth in PLD. In some patients, liver volume decreases after menopause. Female sex hormones could therefore be a target for therapy. The AGAINST-PLD study will examine the efficacy of the GnRH agonist leuprorelin, which blocks the production of estrogen and other sex hormones, to reduce liver growth in PLD.

**Methods:**

The AGAINST-PLD study is an investigator-driven, multicenter, randomized controlled trial. Institutional review board (IRB) approval was received at the University Medical Center of Groningen and will be collected in other sites before opening these sites. Thirty-six female, pre-menopausal patients, with a very large liver volume for age (upper 10% of the PLD population) and ongoing liver growth despite current treatment options will be randomized to direct start of leuprorelin or to 18 months standard of care and delayed start of leuprorelin. Leuprorelin is given as 3.75 mg subcutaneously (s.c.) monthly for the first 3 months followed by 3-monthly depots of 11.25 mg s.c. The trial duration is 36 months. MRI scans to measure liver volume will be performed at screening, 6 months, 18 months, 24 months and 36 months. In addition, blood will be drawn, DEXA-scans will be performed and questionnaires will be collected. This design enables comparison between patients on study treatment and standard of care (first 18 months) and within patients before and during treatment (whole trial). Main outcome is annualized liver growth rate compared between standard of care and study treatment. Secondary outcomes are PLD disease severity, change in liver growth within individuals and (serious) adverse events. The study is designed as a prospective open-label study with blinded endpoint assessment (PROBE).

**Discussion:**

In this trial, we combined the expertise of hepatologist, nephrologists and gynecologists to study the effect of leuprorelin on liver growth in PLD. In this way, we hope to stop liver growth, reduce symptoms and reduce the need for liver transplantation in severe PLD.

*Trial registration* Eudra CT number 2020-005949-16, registered at 15 Dec 2020. https://www.clinicaltrialsregister.eu/ctr-search/search?query=2020-005949-16.

## Background

Polycystic liver disease (PLD) is a rare disease, characterized by the presence of > 10 liver cysts [1]. Autosomal Dominant Polycystic Liver Disease (ADPLD) leads to PLD only and is caused by a mutation in *PRKCSH*, *SEC63*, *LRP5*, *ALG8* or *SEC61B* [1]. Autosomal Dominant Polycystic Kidney Disease (ADPKD) can cause PLD but also leads to kidney cysts and renal function decline [1–3] and is most frequently caused by a mutation in the *PKD1* or *PKD2* gene [4, 5]. The *PKD1* and *PKD2* genes encode for ciliary proteins polycystin 1 and polycystin 2, respectively, while the mutations in ADPLD affect maturation and trafficking of polycystin 1 [3, 4]. The prevalence of ADPLD is < 1:10.000 [6], the point prevalence of clinically relevant ADPKD approximately 1:2500 [7], but not all ADPKD patients suffer from PLD [8].

PLD patients have a normal liver function in general, but large liver volumes can lead to serious physical and psychosocial complaints [1, 9]. The large liver can compress the stomach and bowels, leading to early satiety, decreased food intake, weight loss, sarcopenia and constipation [1, 9]. The increased intra-abdominal volume can lead to pain, and diaphragmatic, umbilical and inguinal herniation [1] and compression of vascular structures can lead to ascites, portal vein thrombosis or hepato-venous outflow obstruction [10]. The large, protruding abdomen may cause psychological problems, because of distorted body image and confronting questions about possible pregnancy [1, 9]. This can result in decreased quality of life, and sometimes liver transplantation is the only and last resort treatment option [11, 12].

### An unmet need for new treatments

**Fig. 1:**
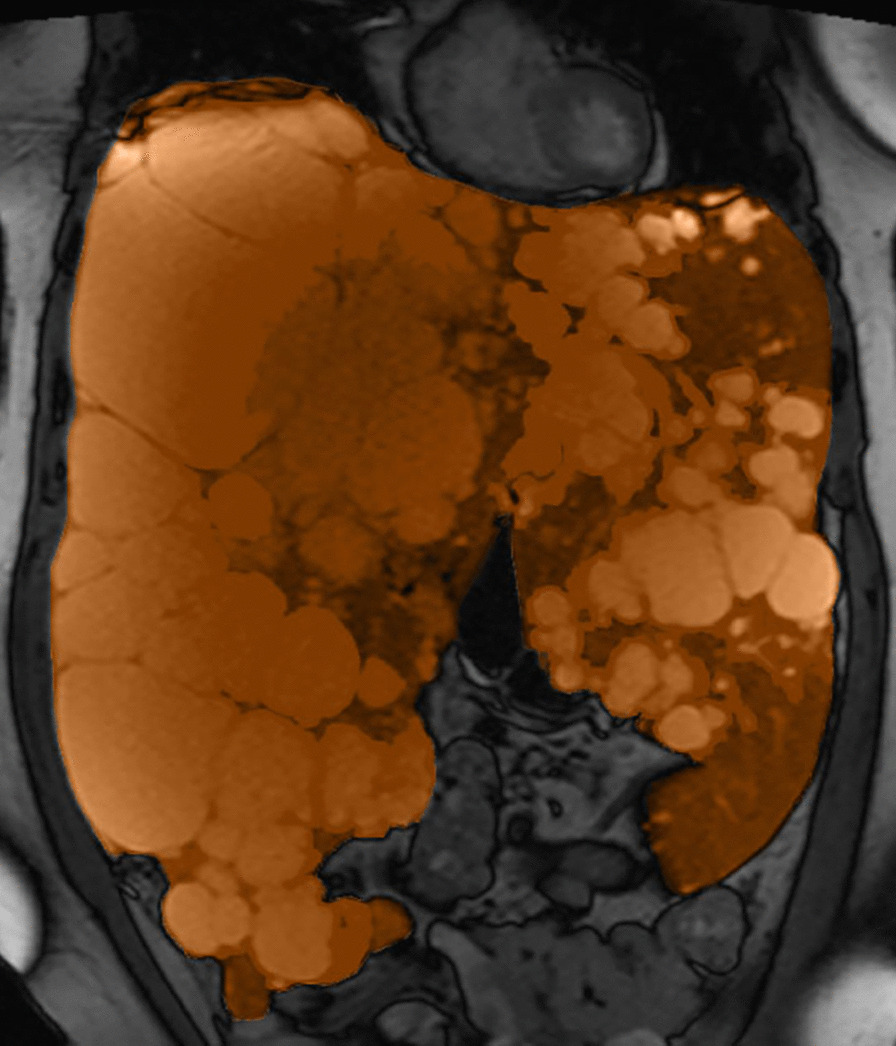
MRI scan (coronal T2-weighted image) of a patient with severe PLD and ongoing liver growth despite somatostatin analogue use, that would be eligible for the AGAINST-PLD study

Dependent on the cyst distribution, different treatments are possible. In case of several dominant liver cysts, aspiration or fenestration is used [1, 9]. If liver cysts are clustered in a few liver segments, a hemihepatectomy can be performed [1]. In case of numerous liver cysts in all segments, surgical interventions are not possible and the mainstay of treatment is to halt natural growth [1, 9]. The only treatment now available to halt growth of liver cysts are somatostatin analogues, but in about 40% of patients treated with these drugs liver growth continues (Fig. 1) [13]. In these patients, the only treatment option that remains is a liver transplantation [1, 9, 12]. In the last 15 years, 1293 liver transplantations have been performed in Europe to treat PLD [12]. Therefore, there is a continuing unmet need for new therapies in PLD.

### Female hormones in PLD

Female sex is the most important risk factor for PLD [14]. About 90% of patients is female [14, 15]. Several factors indicate that female sex hormones stimulate cyst growth. First, estrogen supplementation leads to enhanced liver growth [16, 17] and after menopause, when estrogen and progesterone levels fall, liver volumes sometimes decrease spontaneously [18]. Second, estrogen receptors are present on hepatic cyst epithelium and in vitro, liver growth increases after estrogen supplementation and decreases after administration of estrogen blockers [19–22]. In a recent review paper, we explained in detail how the female sex hormones estrogen and progesterone may affect liver growth and how these hormones could be a target for therapies [22]. While there is a lot of evidence that estrogen affects liver cyst growth, it is difficult to disentangle the contributions of the individual sex hormones, like estrogen and progesterone, on PLD. Oral contraceptives mostly contain estrogen as well as progesterone and menopause also affects both hormones. Therefore, it is unclear whether other sex hormones than estrogen, such as progesterone, affect liver growth.

An ideal therapy should block all estrogen receptors and should block also other sex hormones, such as progesterone. For estrogen only, three different estrogen receptors [22] are present on hepatic cyst epithelium: ER-α, ER-β and the G-coupled protein estrogen receptor 1 (GPER-1) [19–23]. Selective estrogen receptor modulators (SERM), such as tamoxifen, act as agonist or antagonist on ER-α and ER-β, dependent on the surrounding tissue [24, 25]. Currently it is not possible to predict the effects of SERMs in PLD. Besides, the effect on the GPER-1 receptor (inhibition or stimulation) is unclear. Therefore, in this trial, we aim to reduce liver growth in patients suffering from severe PLD using the Gonadotropin Releasing Hormone (GnRH) agonist leuprorelin. This drug leads to cessation of the production of estrogen as well as progesterone and other female hormones, aiming to stop liver growth, but with medically induced menopause as logical adverse event [26].

### Objectives

The main trial objective is:

- To determine whether lowering estrogen and progesterone levels with the GnRH agonist leuprorelin decreases liver growth rates (%/y) in pre-menopausal women with severe PLD when compared to similar women not receiving this treatment.

Secondary objectives are:

1. To assess the effect of leuprorelin on PLD disease severity, measured with the PLD-Questionnaire (PLD-Q)
2. To assess the change in liver growth within individuals before and during leuprorelin treatment
3. To assess the tolerability and incidence of (serious) adverse events.

Exploratory outcomes are:

1. To assess estrogen and progesterone levels
2. To assess differences in short term and long term effects of treatment
3. In case of ADPKD, to assess the effect on kidney growth and renal function decline.

## Methods and design

### Study design

**Fig. 2:**
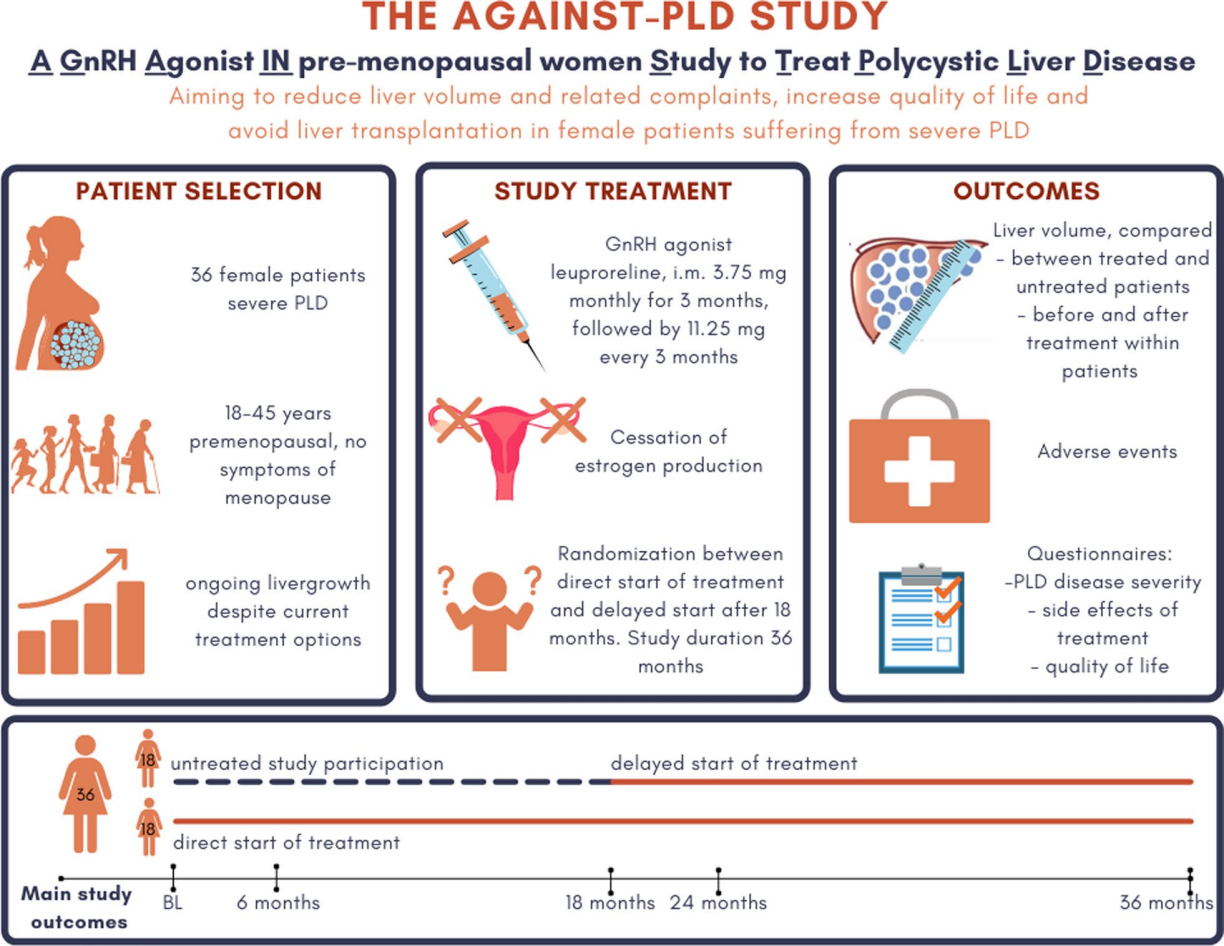
Infographic about the AGAINST-PLD study

**Fig. 3:**
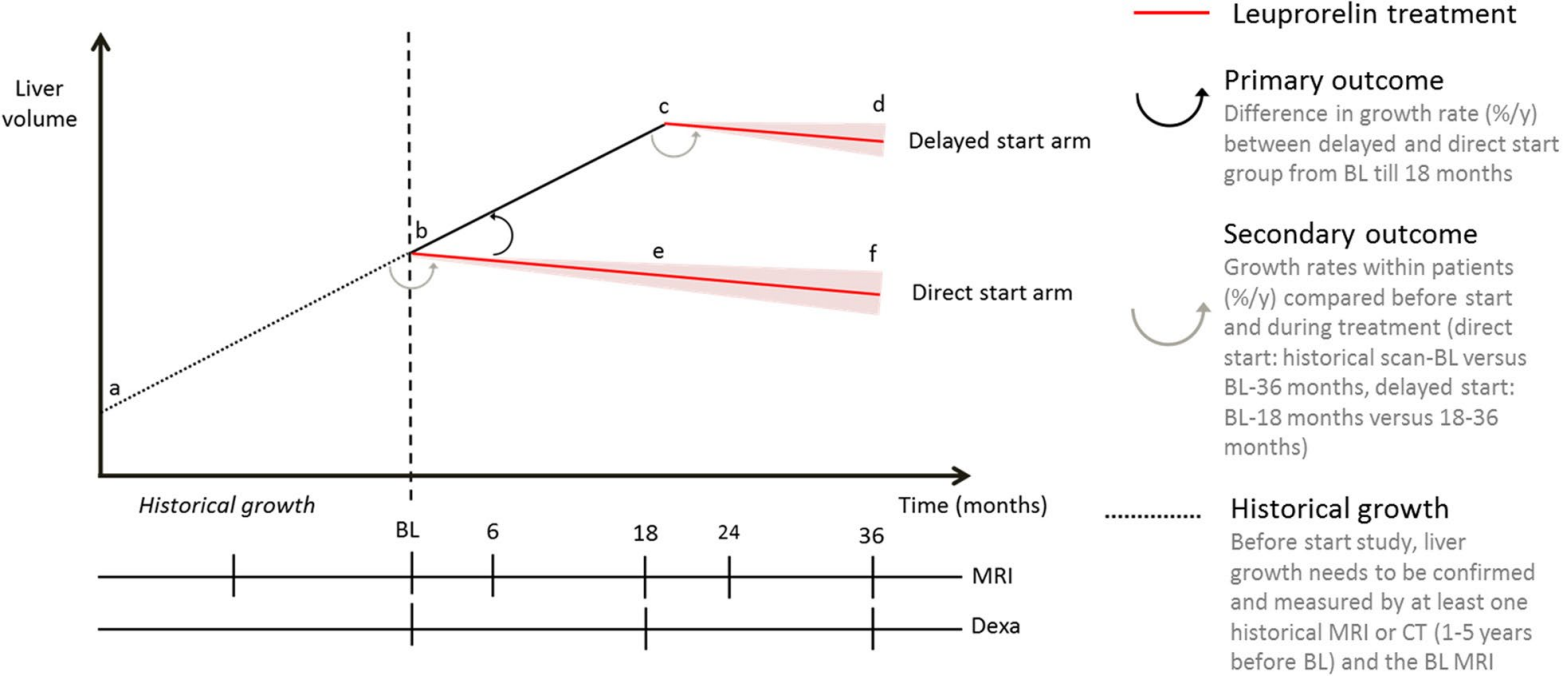
Study design of the AGAINST-PLD study. Patients are randomized between direct and delayed start (after 18 months) of treatment. **a** Historical scan, **b** baseline scan, **a**, **b** historical growth. Primairy outcome, comparison between growth on treatment and without treatment in the first 18 months is **b**, **c** versus **b**, **e**. Secondary outcome, growth within individuals before and during treatment is **b**, **c** versus **c**, **d** and **a**, **b** versus **b**, **e**

The AGAINST-PLD study (A GnRH Agonist IN premenopausal women Study to Treat Polycystic Liver Disease) is a 3-year lasting, investigator driven, phase 2b randomized controlled trial. Participants will be randomized between direct start or delayed start (18 months later) of leuprorelin treatment (Figs. 2, 3). Patients randomized to delayed start will receive standard of care in the first 18 months of the trial. Therefore, in the first 18 months of the trial, we can distinguish between the effect of treatment and the natural course of disease by comparing both treatment arms. Using data obtained during the second 18 months of the trial, when all patients are subjected to active treatment, we can compare liver growth rates within individuals before start of treatment and during treatment and obtain more information about safety and tolerability.

### Study setting

The trial will be conducted according to the International Conference of Harmonization Good Clinical Practice Guidelines and adheres to the Declaration of Helsinki. IRB approval will be obtained in all participating centers. All participants must give their voluntary and written informed consent before any study related procedures could take place. Individuals who meet the entry criteria and complete the baseline visit will be enrolled in one of the five participating University Medical Centers: Groningen and Nijmegen, (the Netherlands), Cologne (Germany), Leuven (Belgium) and Barcelona (Spain).

### Patient selection

Detailed inclusion criteria are given in Table 1, of which the most important are:

- Female patients, pre-menopausal
- Anti Mullerian Hormone (AMH) level > 0.3 (a lower level indicates upcoming menopause)
- Age 18–45 years (inclusive)
- PLD with a very large liver for age, based on the upper 10% of volumes in our PLD registry (n = 1600) [27].
- One historical MRI or CT scan, performed 1–5 years before baseline, available to calculate liver growth before start of the study
- Ongoing liver growth, confirmed using a historical scan (a) obtained in routine clinical care and the scan at screening (b) with the liver volume in ml from scan b–a > 0.
- Use of a somatostatin analogue (only proven effective therapy currently available) or reason not to use such drug (e.g. not tolerated, ineffective, not available)

**Table 1:** Overview of in- and exclusion criteria for the AGAINST-PLD study

Inclusion criteria	
Female	
Diagnosis of PLD (presence of > 10 cysts)	
Age 18–45 (inclusive) years	
Very large liver for age:	
18–30 y hTLV > 2.0 L/m	
30–35 y hTLV > 2.2 L/m	
25–40 y hTLV > 2.5 L/m	
40–45 y hTLV > 3.0 L/m	
1 historical MRI or CT scan of the liver available, made 5-1 y before baseline	
Ongoing liver growth, confirmed using the historical scan and MRI scan at screening	
Use of somatostatin analogues OR a reason not to use them (e.g. tried in de past but stopped because of side effects, not effective, no acces, patient does not want to)	
Voluntary written informed consent	

Exclusion criteria	
Post menopausal status or (vasomotor) symptoms indicating upcoming menopause	
AMH < 0.03 ng/ml at screening	
Active desire to have children, pregnancy or breast-feeding	
Contra-indications for leuprorelin, such as cardiovascular disease or osteoporosis	
Liver transplantation expected within 1.5 years	
Use of oral estrogen or progesterone containing medication	
Contra-indications for MRI	
Chronic use of immunosupressive agents	
Severe hypertension (systolic pressure ≥ 160 and/or diastolic pressure ≥ 100 mmHg)	
Clinically significant uncontrolled medical condition that, in the opinion of the investigator, would put the safety of the patient at risk though participation, or which would affect efficacy or safety analysis, such as, but not limited to, recurrent cholangitis, recurrent ascites, hepato-venous outflow obstruction, (history of) depression	
Participation in other interventional studies at the same time	

Exclusion criteria related to the historical MRI/CT scan	
Start or stop of liver volume reducing therapy (medication e.g. somatostain analogues or surgical interventions) between the historical scan and screening	
Historical scan is performed < 3 months after start or stop of volume reducing therapy (either a somatostatin analogue or surgical treatments)	

Most important exclusion criteria are:

- Symptoms of (upcoming) menopause, e.g. hot flushes
- Active desire to have children during study period
- Use of exogenous estrogen and/or progesterone
- Contra-indication for leuprorelin

### Randomization

Participants will be randomized 1:1 between direct and delayed start (after 18 months) of treatment using an online Interactive Response System (IXRS) tool, stratified for somatostatin analogue use (yes/no) and age (< 40 and ≥ 40 years).

### Intervention

Leuprorelin is a synthetical analogue of GnRH and strongly stimulates the pituitary resulting in desensitization. Thus, the production of luteinizing hormone (LH) and follicle stimulating hormone (FSH) by the pituitary stops, which leads to cessation of the production of estrogen and progesterone by the ovaries.

Leuprorelin will be started using monthly injections of 3.75 mg, and if tolerated after 3 months, treatment regimen will be switched to 3-monthly injections of 11.25 mg. Depending on patients’ preferences, participants will be trained to self-administer the injections at home or injections will be administered by study personnel.

### Concomitant medication

For massive PLD, somatostatin analogues are currently the only evidence-based treatment. Therefore, somatostatin analogues should be used, tried, or have been considered before inclusion in the AGAINST-PLD trial. Patients are thus allowed to use a somatostatin analogue during the trial (see inclusion criteria, Table 1). During the study, start or stop of somatostatin analogues as well as surgical procedures to reduce liver volume should be avoided unless there are important reasons to do so, because this could interfere with the assessment of the primary outcome, liver growth.

Use of estrogen- and or progesterone containing medication is not allowed during the trial period, because it could interfere with the effect of the study treatment. Blood pressure treatment is targeted below 130/80 mmHg with RAAS-inhibitors as the first-choice agents. Calcium vitamin D (500 mg/800 IE) will be prescribed to all patients in the study to prevent osteoporosis.

### Outcome parameters

#### Primary outcome

- Liver growth (%/y), calculated over the first 18 months of the trial and compared between the direct start group (on treatment) and delayed start group (on standard of care), b–e versus b–c, Fig. 3.

#### Secondary outcomes

1. Change in PLD disease severity, assessed by the validated PLD-Q questionnaire, calculated over the first 18 months of the trial and compared between the direct start group (on treatment) and delayed start group (on standard of care).
2. Liver growth rates within individuals compared before treatment and during treatment. In the direct start group, historical growth rates (Fig. 3a, b) will be compared with growth rate during 18 months of treatment (b–e) and in the delayed start group, growth rates during the first 18 months of the trial (untreated, b–c) will be compared with the last 18 months of the trial (treated, c–d).
3. The incidence of (serious) adverse events, compared between the direct and delayed start group in the first 18 months of treatment.

#### Exploratory outcomes

- Height adjusted liver volumes, compared between the direct and delayed start group after 18 months
- Estradiol, progesterone, AMH, LH and FSH levels at baseline, 6, 18, 24 and 36 months
- Difference between acute and chronic effects of treatment on liver growth as assessed by a mixed models analysis comparing liver growth rate in the first 6 months of treatment compared to the next 12 months of treatment.

With respect to tolerability:

- Quality of life, using the validated 36-Item Short Form Health Survey (SF-36) and the Beck Depression Inventory II (BD-II) questionnaires
- Menopause related complaints: using the Menopause Specific Quality of Life (MENQOL) questionnaire
- Bone density: T-score on Dual Energy X-ray Absorptiometry (DEXA) scans will be compared between direct start group and delayed start group after 18 months.

For patients with polycystic kidney disease (PKD) also:

- Kidney growth (%/y) calculated over the first 18 months of the trial compared between direct and delayed start group
- Kidney growth rates within individuals compared before treatment and during treatment
- Differences between acute and chronic effects of treatment on kidney growth
- Renal function decline before and during treatment.

### Sample size calculation

In our own DIPAK 1 trial [13], the mean liver growth rate in patients meeting the inclusion criteria was + 5.8% ± 2.8% per 18 months. Since the information on liver growth rate in this specific group is scarce, we conservatively doubled the SD to ensure enough power. We aim at fully stopping liver growth. Using β = 80%, α = 0.05, and incorporating a 20% drop-out rate, inclusion of 36 patients is needed to show this effect. In other proof-of-concept studies in PLD, similar numbers of patients were included [28, 29].

### Recruitment

Patients will be recruited from outpatient clinics of the participating centers or referred by other centers. All participating centers are expertise centers on polycystic liver and/or polycystic kidney disease and see many patients that meet the inclusion criteria for the trial. We planned 24 months for the recruitment period.

## Data collection and management

**Fig. 4:**
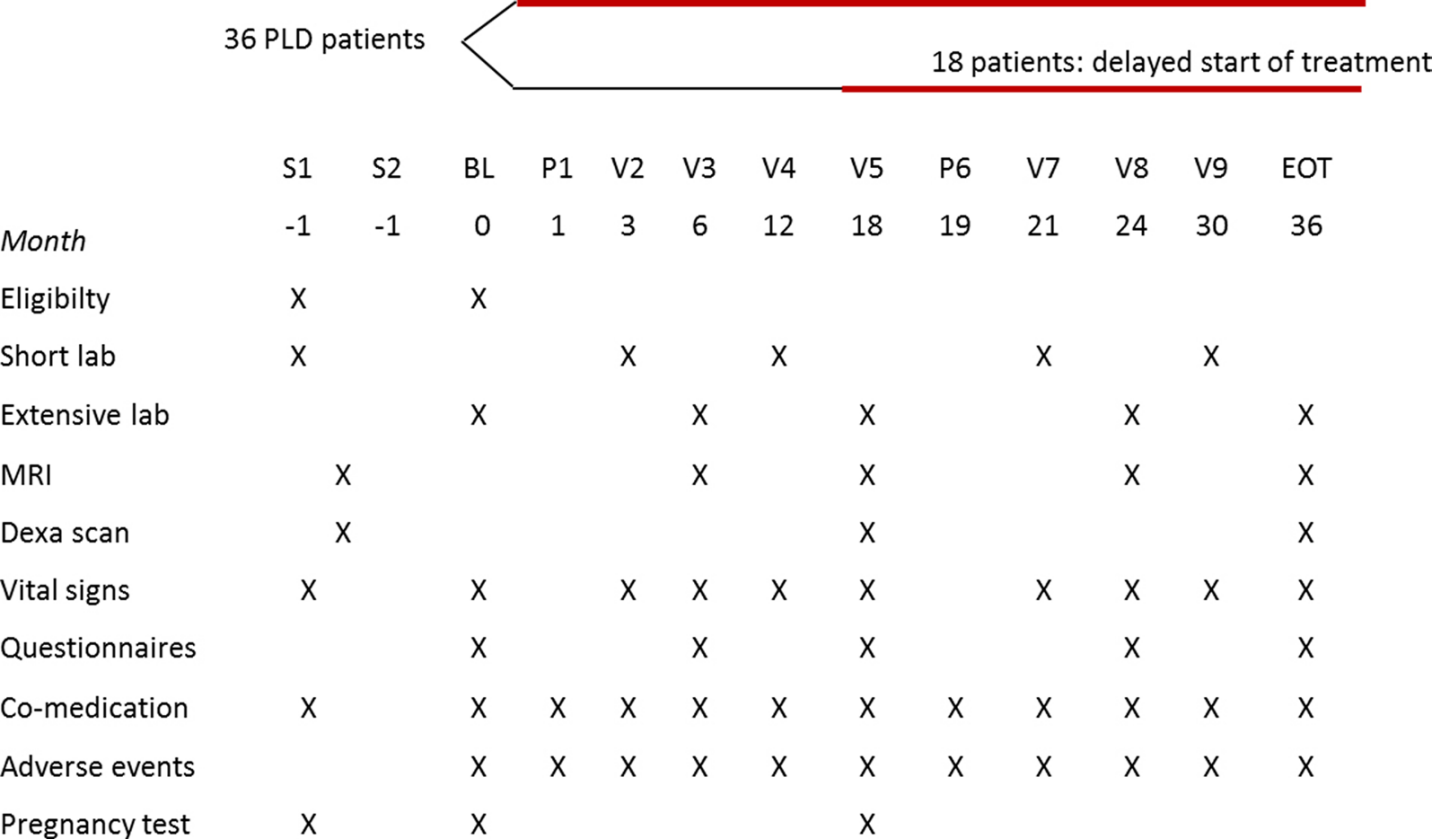
Visit schedule. S = screening, BL = baseline, V = visit, P = phone call. Screening procedures can take place in one visit or with MRI and DEXA apart, as preferred by site and patient. Patients are randomized to direct start (at BL) or delayed start (at V5). A phone cal (P) takes place one month after first leuprorelin injection. More extensive visits with MRI, questionnares and extensive lab take place at BL, 6, 18, 24 and 36 months. Extensive lab consists also of biobanking (samples stored at − 80 °C and shipping to core laboratory for measurement of female sex hormones in one run) and in case of ADPKD, 24 h urine collection. Between visits, site personnel will remind patients to administer the leuprorelin injections by phone or email

The study consists of one or two screening visits, nine hospital visits and one phone call. During short visits (3, 12, 15, 21 months), a small set of local laboratory measurements, vital signs, AEs and concomitant medication will be assessed. At larger visits (BL, 6, 18, 24 and 36 months) in addition MRI scans, extensive lab and questionnaires will be performed. DEXA scans are made at baseline, halfway and end of the study (Figs. 3, 4). Data will be entered in a web-based electronic case report form to ensure correct and timely data collection in a central database.

### MRI scans

Liver and kidney volumes (the latter in ADPKD patients only) will be measured using an MRI scan at screening, 6, 18, 24 and 36 months. MRI scans will be performed using a standardized protocol, including a coronal T2-weighted single-shot fast gradient spin-echo sequence with fat-saturation with a slice thickness of 3 mm which is the primary sequence to determine liver volumes. In addition, a transversal T2-weighted single shot fast gradient spin-echo sequence with fat suppression and a coronal T1-DIXON-VIBE spoiled gradient echo sequence will be obtained. After pseudonymization, MRI scans will be sent to the central reading facility, where images will be analyzed blinded for patient, time and treatment by trained observers using an automated MRI program [30]. To ensure that valid MR images are obtained, quality control will be performed within 72 h by trained personnel. In case a scan is rejected, it will be repeated.

### Laboratory measurements

For a detailed overview of collected samples, see Fig. 4. At all visits, a limited set of laboratory measurements will be performed. At baseline and the visits at month 6, 18, 24 and 36, more extensive laboratory measurements, biobanking and in ADPKD patients, 24-h urine samples will be collected. Except form biobanking samples, laboratory samples will be analyzed locally. Biobanking samples will be stored at − 80 °C and shipped to the core laboratory at the end of the trial to measure estrogen, progesterone, AMH, FSH and LH in one run, to minimize interlaboratory and interassay variation.

### Vital signs

During every visit we will measure blood pressure, weight and abdominal circumference. Height will be collected at baseline. Upper arm circumference of the non-dominant arm will be measured every visit because this is one of the exception criteria for the MELD score [31].

### Questionnaires

Questionnaires will be sent digitally to the patient at screening, 6, 18, 24 and 36 months. Quality of life will be assessed using the SF-36 and BD-II questionnaires, PLD disease severity using the validated PLD-Q questionnaire and menopause related complaints using the MENQOL questionnaire [32, 33].

### Criteria for withdrawal

Subjects can leave the study at any time for any reason if they wish to do so without any consequences. Patients that drop-out from the trial within the first 3 months from baseline will be replaced and not be part of the intention to treat analysis. Patients that drop-out from the trial later on will not be replaced by new patients and will be asked for an early-end of treatment. The investigator can withdraw a subject from the study for urgent medical reasons, such as (but not limited to): occurrence of a concomitant disease in which leuprorelin is contra-indicated, pregnancy, need of treatment that is contra-indicated during the trial (such as estrogens), lost-to-follow up or, by the opinion of the treating physician, high need for surgical volume reducing treatments. If a patient that is registered on the waiting list receives a transplant offer, this patient will also be withdrawn from the trial.

### Statistics

#### Statistical analysis

Analyses are performed after completion of the study. For the primary outcome, liver growth during the first 18 months of the trial compared between direct and delayed starters, an unpaired t-test will be used. In case of missing data, last information will be used adjusted for the duration of follow up. For questionnaires, the delta in scores during the first 18 months of the trial will be compared between direct and delayed starters. To compare liver growth within individuals before and during treatment, a paired t-test will be used. Adverse events will be categorized and the number of adverse events per category will be compared between direct and delayed starters using an unpaired t-test.

For all primary and secondary analyses, we will perform pre-defined sensitivity analysis to adjust for age, disease type (ADPKD vs. ADPLD), total liver volume at baseline, total kidney volume at baseline, use of somatostatin analogues, and test differences in treatment effects among these variables (interaction). In a sensitivity analysis we will test for differences in the outcome variables between the first 18 months of the trial and the second 18 months of the trial in the direct start group, using a breakpoint analysis. All analyses will be performed based on an intention to treat principle. As a sensitivity analysis, we will perform a per-protocol analysis.

In an additional analysis we will explore whether liver growth is linear and whether the liver growth rate during treatment is different in the first 6 months of treatment compared to the 12 months thereafter, using a multiple mixed model.

## Discussion

The AGAINST-PLD study will assess the effect of the GnRH-agonist leuprorelin on liver growth in pre-menopausal patients with severe PLD. If effective, leuprorelin may help to stop disease progression in female PLD patients at risk of rapid disease progression. Ultimately, this may avoid liver transplantations, which is desirable in view of the donor shortage and the procedure related morbidity and mortality [12].

We study the effect of anti-estrogenic therapy in PLD, a novel and promising target, using a broad proof-of-concept approach. To reiterate a recent literature review [22], estrogens have a cyst growth promoting effect and the idea to study estrogen inhibition in female PLD patients has been coined several times [3, 20, 22, 34]. To inhibit the effect of estrogens, several approaches could be used [22]. We chose for a GnRH-agonist, because this is the only way currently available to inhibit all three types of estrogen receptors and also block the effects of other female hormones such as progesterone. For other treatments, such as tamoxifen (a selective estrogen receptor modulator) it is so far unclear whether they would inhibit, stimulate or either have no effect on estrogen receptors in PLD and they would not block other female hormones which possibly also affect liver growth [22].

Since the study treatment ceases the menstruation and can lead to menopause-related complaints, blinding is not possible and as a consequence we adopted a prospective open-label with blinded endpoint assessment (PROBE) design. Randomization between direct and delayed start of treatment makes it possible to compare treated and untreated patients, but also to compare liver growth before and during treatment within patients, and it will render additional safety and tolerability data. Finally, the design meets the urgency felt by patients, stating that they would not participate if they could be in a control group without treatment.

### Efficacy–safety ratio

Leuprorelin blocks the production of all female sex hormones and can have several side effects that we know from menopause, such as hot flushes, night sweats, mood swings, vaginal dryness and disturbed sleep [26]. On the long term, estrogen depletion could increase the risk of osteoporosis and cardiovascular events [35, 36].

We tried to enhance the tolerability and the efficacy-safety ratio of the study medication in several ways. First, only patients with the highest need for new treatment options are included in this study. The target population consist of patients who, despite current treatment options, will mostly need a liver transplantation in the future if the course of the disease cannot be altered. We discussed the design of this trial in a focus group with eight patients meeting the inclusion criteria and with de Dutch Liver Patient Foundation. They felt that the burden of the disease is high and the perspective grim, and most patients thought that the potential of efficacy of a new treatment outweighs side effects.

Second, with the help of patient focus groups we adjusted the study design to the preferences and needs of patients to be included, especially to limit adverse events related to the mechanism of action of GnRH agonists. A potential option to reduce side effects would be to add a low-dose estrogen and/or progesterone as “add-back therapy”, as is used in GnRH agonist treatment for endometriosis [26]. However, because this is a proof-of-concept trial testing whether estrogen depletion curtails liver growth, we decided to not allow add-back therapy. Instead, we added that non-hormonal treatments can be used to treat side effects, such as clonidine in case of hot flushes and venlafaxine in case of mood swings. In addition, also at the request of the patients in the focus group, a gynecologist will be available at all study sites to counsel patients before and during the study, at some sites supported by a trained nurse as “menopause coach”.

On the longer term, estrogen depletion could lead to an increased risk of osteoporosis and a slightly enhanced cardiovascular risk [26, 35, 36]. With a treatment duration of 18 or 36 months, we do not expect large detrimental effects on the long term. However, to ensure optimal safety, patients with a history of cardiovascular disease and a low bone-density at screening will be excluded from the trial and during the trial blood pressure, cholesterol levels and bone-density will be monitored (Fig. 4). In addition, all patients will receive advise on healthy food intake and calcium and vitamin D supplementation.

### Interim analysis

To safeguard a favorable efficacy–safety ratio during the trial, a Data Safety and Monitoring Board will meet regularly to discuss the progress and interim results. A formal interim analysis for safety and futility will be performed when 50% of the patients (n = 18) have reached 18 months, the time point at which the primary outcome is assessed.

### Perspective

In case the trial is positive, leuprorelin treatment will at first be especially indicated in patients suffering from severe, symptomatic PLD given the nature of the intervention and the expected side-effects. However, when more experience is gained, and leuprorelin also shows a good efficacy–safety ratio in a real life setting, the use of this drug could be extended to patients with less severe forms of PLD, that notwithstanding are symptomatic.

With the AGAINST-PLD study, we venture outside the somatostatin analogue era and explore a promising new target for intervention to stop PLD progression: inhibition of female sex hormones. In this trial we will include patients with the highest need for treatment. In this way we hope to be able to reduce liver growth, and associated symptoms, an urgent unmet need in PLD.

## Data Availability

De-identified individual participant data collected during the trial will be made available upon request to researchers who provide a methodologically sound proposal and whose use of the data has been approved by the AGAINST-PLD Steering Committee.

## Abbreviations

ADPKD: Autosomal Dominant Polycystic Kidney Disease
ADPLD: Autosomal Dominant Polycystic Liver Disease
AMH: Anti Mullerian Hormone
BD-II: Beck Depression Inventory Questionnaire
CT: Computed tomography
DEXA: Dual Energy X-ray Absorptiometry
DIPAK: Developing Intervention strategies to halt Progression of Autosomal Dominant Polycystic Kidney Disease
ER: Estrogen receptor
FSH: Follicle stimulating hormone
GnRH: Gonadotropin receptor hormone
GPER: G-coupled protein estrogen receptor
LH: Luteinizing hormone
MENQOL: Menopause Specific Quality of Life
MRI: Magnetic resonance imaging
PLD: Polycystic liver disease
PLD-Q: Polycystic Liver Disease Questionnaire
PROBE: Prospective open label study with blinded endpoint assessment
